# Gene Family Expansion during the Adaptation of *Colletotrichum gloeosporioides* to Woody Plants

**DOI:** 10.3390/jof9121185

**Published:** 2023-12-11

**Authors:** Fanli Meng, Chengming Tian

**Affiliations:** 1 The Key Laboratory for Silviculture and Conservation of Ministry of Education, College of Forestry, Beijing Forestry University, Beijing 100083, China; mengfanli@bjfu.edu.cn; 2 Beijing Key Laboratory for Forest Pest Control, College of Forestry, Beijing Forestry University, Beijing 100083, China

**Keywords:** *Colletotrichum gloeosporioides*, host plants, signal recognition, gene family expansion, glycosyde hydrolase family

## Abstract

Gene gains/losses during evolution are critical for the adaptation of organisms to new environments or hosts. However, it remains unknown whether gene family expansions facilitated the adaptation of phytopathogenic fungi to woody plants. In this study, we compared the newly sequenced genome of the *Colletotrichum gloeosporioides* strain CFCC80308 with the genomes of two other *C. gloeosporioides* strains, Cg-14 and Lc-1, isolated from *Persea americana* and *Liriodendron* leaves, respectively. The genes in the expanded families, which were associated with plant surface signal recognition, encoded various proteins, including glycosyde hydrolases (GHs) and cytochrome P450. Interestingly, there was a substantial increase in the number of GH family genes in CFCC80308. Specifically, there were 368 enriched genes in the GH families (e.g., GH1, GH3, GH10, GH12, GH15, GH16, GH17, GH18, GH25, GH32, GH53, GH61, GH76, and GH81); the expression levels of these genes were highly up-regulated during the infection of poplar trees. Additionally, the GH17 family was larger in CFCC80308 than in *C. gloeosporioides* strains Cg-14 and Lc-1. Furthermore, the expansion of the MP65-encoding gene family during the adaptation of *Colletotrichum* species to woody plants was consistent with the importance of gene gains/losses for the adaptation of organisms to their environments. This study has clarified how *C. gloeosporioides* adapted to woody plants during evolution.

## 1. Introduction

The genus *Colletotrichum* consists of more than 190 accepted species, including economically important phytopathogenic fungi that are among the top 10 fungal pathogens that infect plants [1,2]. These fungi, which co-evolved with herbs and woody plants, may have adapted to various hosts. Species evolve and adapt to the conditions of novel habitats, including new hosts [3]. During the last few decades, research on the evolution of species responses to environmental changes [4] has revealed the evolutionary importance of gene gains and losses, especially in terms of gene family dynamics [5]. *Colletotrichum* fungi have a broad host range. Moreover, the expansion of gene families has been detected among these fungal species [6]. It was also reported that spatial organization allows for position effects to stabilize RNA expression and balance transcription, which can be advantageous for a number of reasons, including reductions in stochastic influences between the gene products and the organization of co-regulated gene families into functional clusters occurs extensively in *Dikarya* fungi [7,8,9]. The functionally clustered genes localize to genomic loci that are more conducive to transcriptional regulation at a distance compared to the unpaired members of the same families in *Saccharomyces cerevisiae* [10,11].

The phytopathogenic fungus *Colletotrichum gloeosporioides* is the causal agent of poplar anthracnose and many other foliage diseases, which are responsible for considerable economic losses worldwide. The foliar infections caused by *C. gloeosporioides* are initiated by the adhesion of conidia to the cuticle of leaves, after which the conidia germinate, with a germ tube generated on one side of the spore. The differentiation of the germ tube tip results in the production of a dome-shaped appressorium. In addition to infecting poplar, *C. gloeosporioides* can also infect various economically valuable tree species, such as *Juglans regia*, *Mangifera indica*, *Hevea brasiliensis*, *Camellia oleifera Citrus* spp., and *Cunninghamia lanceolata*, leading to the rotting of leaves, branches, or fruits and substantial decreases in agricultural and forestry production [12,13]. Gene family expansion during adaptive evolution is the main source of genes with novel functions in organisms [14,15]; however, it is unknown whether gene family expansions contributed to the adaptation of *C. gloeosporioides* to new hosts. The expansion of gene families might have been one of the consequences of the adaptation of *C. gloeosporioides* to woody plants.

The appressorium is the main structure mediating the infection of susceptible plants by many phytopathogenic fungi, including the pathogens causing anthracnose, rice blast, rust, and corn smut [12,16,17,18,19,20]. Therefore, exploring the molecular basis of appressorial cell formation and attachment to the host is crucial for elucidating the mechanism underlying pathogenicity. Accordingly, it has become a major research topic in the field of molecular plant pathology. It was reported that pathogenic fungi may be seen to have tuned these basic processes, present in all fungi, to sense the proximity of living plant tissue and respond in a way that promotes colonization. Beyond this, differences in host species or different organs of the same host species can provoke different responses [21]. However, the molecular mechanism of *C. gloeosporioides* through which conidia perceive various woody plant components and features, including hydrophobicity, surface hardness, leaf wax, and keratinocytes, and activate downstream signaling pathways in cells prior to the formation of the appressorium remains unclear.

To explore the genetic basis of the adaptation of phytopathogenic fungi to woody plants, the genome of a *C. gloeosporioides* strain isolated from *Populus beijingensis* was sequenced and compared with the previously sequenced genomes of the Cg-14 and Lc-1 strains [22,23], which were isolated from *Persea americana* and *Liriodendron chinense* leaves, respectively. The findings of this study are consistent with the gene family expansion in *C. gloeosporioides* during the adaptation with woody plants. Furthermore, the study results provide useful insights into the mechanism underlying the adaptive evolution of a phytopathogenic fungus that allows it to infect its host.

## 2. Materials and Methods

### 2.1. Fungal Isolates and Nucleic Acid Extraction

*Colletotrichum gloeosporioides* strain CFCC80308 (China Forestry Culture Collection Center 80308) was isolated from a lesion on a leaf of an infected *P. beijingensis* tree growing in Beijing, China. Axenic cultures were deposited in the China Forestry Culture Collection Center (CFCC). The strain was cultured on potato dextrose agar medium in plates that were incubated at 25 °C. Because the colonies growing for 5 days produced a large number of conidia and germinated well, the conidia were harvested from 5-day-old cultures. Conidial suspensions were obtained by washing them three times in sterile water and centrifugating them to collect samples. Genomic DNA was extracted from *C. gloeosporioides* using the TRIzol reagent (Invitrogen, Carlsbad, CA, USA) according to the manufacturer’s protocol. Samples were stored at −80 °C. Genomic DNA concentrations were determined using the NanoDrop ND-8000 spectrophotometer (NanoDrop, Waltham, MA, USA). The quality of the DNA was assessed by agarose gel electrophoresis.

### 2.2. Genome Sequencing, Assembly, and Validation

The *C. gloeosporioides* CFCC80308 genomic DNA was sequenced using the Illumina Genome Analyzer X Ten and the PacBio Sequel System. Raw reads were processed using custom Perl scripts to trim adapters and eliminate low-quality reads and contaminated nucleotides. After removing the PacBio sequencing adapters and primers, the CCS HiFi reads were assembled to *C. gloeosporioides* contigs using Hifiasm software (version 0.13) [24]. The filtered reads were assembled using the Platanus software (version 1.2.1) (starting size: 25-mer) [25]. Scaffold gaps were closed using the GapCloser software (version 1.12-r6) [26]. The draft genome assembly was polished using Illumina short reads for indel and error correction to generate a final high-quality assembly by Pilon software (version 1.24) [27]. The completeness of the genome assembly was evaluated using BUSCO (version 3.0.2) and Ascomycota data [28].

### 2.3. Gene Prediction and Annotation

Protein-coding genes were predicted using homology-based and ab initio prediction methods. For the homology-based prediction, proteins from Uniprot were mapped onto the genome (repeat-masked) using SPALN software (v2.0.6) [29]. For ab initio prediction, AUGUSTUS software (v3.0.1) [30] and GlimmerM software (v3.0.2) [31] were used to predict gene model. For the transcriptome-based predictions, RNA-seq was mapped to genome to construct transcriptome-based gene models by PASA, and ORFs were extracted by PASA’s inner-built Transdecoder program. They were then merged using EvidenceModeler (version 1.1.1) [32] and refined using custom Perl scripts. All predicted proteins served as queries for the BLASTP search of the NCBI NR database and the UniProt database (E-value < 1 × 10^−5^ and sequence identity > 30%). Motifs and domains were identified, and enriched Gene Ontology (GO) terms were determined using InterProScan [33]. The KO terms were assigned using the Kyoto Encyclopedia of Genes and Genomes Automatic Annotation Server [34]. Secreted proteins were predicted using SignalP [35], whereas proteins potentially associated with pathogenicity were predicted using PHI-base (i.e., pathogen–host interaction database) [36].

### 2.4. Gene Family Construction and Phylogenetic Analysis

Gene families were identified on the basis of the protein sequences of three *C. gloeosporioides* strains (CFCC80308, Cg-14, and Lc-1) and other *Colletotrichum* species by OrthoMCL (v2.0.9) clustering algorithm [37]. A maximum likelihood phylogenetic tree inferred from the concatenated protein data was constructed using RA × ML (version 8.0.24) [38], with 200 bootstrap replicates and the best-fit model (LG + I + G + F) determined by ProtTest (version 3.4).

### 2.5. Gene Family Analysis

The phylogenetic tree file from RA × ML and the binary profile from OrthoMCL were analyzed using the DOLLOP program from the PHYLIP package (version 3.695) to determine gene gains and losses for all species. The gene family data obtained from DOLLOP were analyzed further using HMMScan (version 3.0) [39] to screen for domains in the Pfam database. The genes with no domain hits were eliminated.

### 2.6. Identification of the Genes Related to the Recognition of Plant Surface Signals and Analysis of Gene Co-Expression

To investigate the expansion of *C. gloeosporioides* gene families during the adaptation with the woody plants, we focused on the genes associated with the recognition of plant surface signals. *Colletotrichum gloeosporioides* gene expression data were downloaded from the NCBI SRA database. Reads were trimmed using Trimmomatic (version 0.36) [40] and then mapped onto the CFCC80308 reference sequence using STAR (version 2.5.3a) [41]. The read counts for each gene were calculated using featureCounts (version 1.6.2) [42], whereas gene expression levels were determined using DESeq2 [43]. A co-expression network was constructed using the WGCNA package. A GO enrichment test was performed using the topGO package [44], with the results visualized using the pheatmap package [45].

### 2.7. Transcription Sequencing and Identification of Differentially Expressed Genes

We inoculated *P. beijingensis* leaves with *C. gloeosporioides* strain CFCC80308. Conidia were diluted in sterile water to 2 × 10^5^ conidia/mL and then incubated on the *P. beijingensis* leaves. Samples of the inoculated material were collected at the following time intervals: 0 h (conidia), 3 h (germ tube), 5 h (initial appressorium), 7 and 9 h (gradually maturing appressorium), and 12 h (mature appressorium). Total RNA was extracted from the samples for the analysis of the *C. gloeosporioides* transcriptome during the infection of *P. beijingensis* leaves [46]. Samples were stored at −80 °C. Total RNA was extracted from the ground material using TRIzol reagent (Invitrogen) [47]. The integrity and quantity of the extracted RNA were determined using the 2100 Bioanalyzer (Agilent). High-quality RNA samples were retained for RNA-seq analysis. At least 3 μg total RNA was used for each sample. Gene abundance levels were estimated using the formula TPM (transcripts per million) [48]. Three biological replicates were used, and the differentially expressed genes (DEGs) (with a threshold of FDR value <0.05 and fold change >1) were obtained using the R package DEseq.

### 2.8. Quantitative Real-Time PCR (qRT-PCR)

The expression of key DEGs was analyzed by qRT-PCR. Total RNA was reverse transcribed using the Hifair^®^ II 1st Strand cDNA Synthesis SuperMix for qPCR (with gDNA digester plus) (Yeasen Biotechnology, Shanghai, China). The qRT-PCR analysis was performed using the CFX Connect Real-Time PCR instrument (Bio-Rad, Fort Worth, TX, USA) and the Hieff UNICON^®^ Universal Blue qPCR SYBR Green Master Mix (Yeasen Biotechnology). The *Cg18S* gene served as an internal reference in this study. Relative gene expression was calculated using the 2^−∆∆Ct^ method [49].

### 2.9. Data Deposition

The CFCC80308, Cg-14, and Lc-1 genome sequence data have been deposited in GenBank (accession numbers: PRJNA961031, PRJNA176412, and PRJNA577812). All transcriptome data have been deposited in National Center for Biotechnology Information (NCBI) under accession codes of PRJNA1020205 (BioSample ID: SAMN37517800–SAMN37517818).

## 3. Results

### 3.1. Genome Assembly, Annotation, and Assessment of Genetic Diversity

The Hifiasm software was used to assemble ccs reads, after which errors in the assembled genome were corrected using the Pilon software and second-generation data to obtain a highly accurate final genome. The assembled CFCC80308 draft genome comprised 60.7 Mb (N50 = 5.25 Mb) with approximately 80× coverage (Table 1). The Cg-14 and Lc-1 genome assemblies consisted of 53.2 Mb (N50 = 25 kb) and 61.9 Mb (N50 = 0.71 Mb), respectively.

### 3.2. Evolution of Gene Families

**Figure 1:**
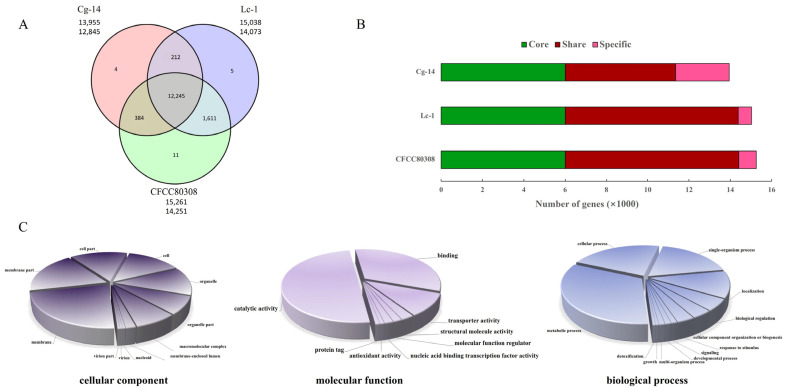
Analysis of the gene families in three *C. gloeosporioides* strains. (**A**) Venn diagram presenting the number of genes in different strains (CFCC80308, Lc-1, and Cg-14). (**B**) Gene distribution in different strains (CFCC80308, Cg-14, and Lc-1). (**C**) GO terms assigned to the genes unique to CFCC80308.

The evolution of three *C. gloeosporioides* strains was investigated, with a particular focus on the adaptation with woody plants. Gene gains and losses are important evolutionary processes enabling organisms to adapt to their environment. The OrthoMCL software was used to classify gene families according to the predicted protein sequences of the sequenced strains and the protein sequences encoded by the reference genome. The gene families were subsequently analyzed to identify and characterize the gene families that were common to all strains and those that were specific to a particular strain. The results of the gene family analysis were visualized in a Venn diagram. Additionally, the gene families were functionally annotated using the Pfam database (Figure 1A,B, Tables S1 and S2). A total of 16,075 genes and 15,261 gene clusters were detected in strain CFCC80308. Moreover, of the 14,251 identified gene families in CFCC80308, 11 were unique to this strain, such as microsomal signal peptidase, amino acid kinase, sodium/calcium exchanger protein, and ATP synthase. The enriched KEGG metabolic pathways and GO terms (Figure 1C) among the genes specific to CFCC80308 were determined. There were 33% unique genes of CFCC80308 enriched in the metabolic process. The main enriched KEGG metabolic pathways were glycosylphosphatidylinositol (GPI)-anchor biosynthesis (ko00563; Figure S1) and glycerophospholipid metabolism (ko00564; Figure S2).

### 3.3. Gene Family Expansion and Evolution of Plant Surface Signal Recognition

Many of the gene families that expanded during evolution were involved in plant surface signal recognition. The genes in the genomes of *C. gloeosporioides* strains CFCC80308, Cg-14, and Lc-1 included those encoding cytochrome P450 (CYP450), glycosyde hydrolases (GHs), carbohydrate-active (CAZy) enzymes, G protein-coupled receptors (GPCRs), dehydrogenases, transcription factors, soluble N-ethylmaleimide sensitive factor attachment protein receptor (SNARE), common in several fungal extracellular membrane (CFEM) proteins, Src homology region 3 (SH3) proteins, and heterokaryon (HET) proteins (Table 2). Although the three analyzed *C. gloeosporioides* strains had a similar number of genes in the expanded gene families (Table 2), we focused on the number of genes in the CFCC80308 genome, which was the most completely assembled and annotated genome in this study.

**Figure 2:**
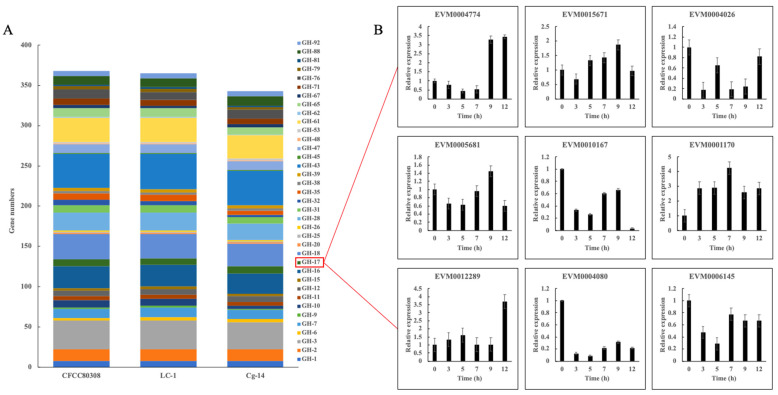
Expansion of glycosyde hydrolase gene families in *Colletotrichum gloeosporioides*. (**A**) Number of glycosyde hydrolase family genes in different strains (CFCC80308, Lc-1, and Cg-14). (**B**) Expression of glycosyde hydrolase family 17 genes at different time-points (0, 3, 5, 7, 9, and 12 h).

According to the KEGG metabolic pathway-based functional annotation, there was a substantial increase in the number of GH family genes in CFCC80308. There were 368 enriched genes in the GH families, including GH1, GH2, GH3, GH7, GH10, GH16, GH17, GH18, GH28, GH31, GH35, GH43, GH47, GH61, GH65, GH71, GH76, and GH88 (Figure 2A). There were considerably more GH17 family genes in CFCC80308 than in Cg-14 or Lc-1. We analyzed the expression of the GH17 family genes at the following time points corresponding to specific stages of appressorium formation: 0 h (conidia), 3 h (germ tube), 5 h (initial appressorium), 7 and 9 h (gradually maturing appressorium), and 12 h (mature appressorium). The gene expression data indicated that the gene encoding the cell surface mannoprotein MP65 (EVM0004774) was highly expressed at 9 and 12 h (Figure 2B), indicative of the importance of the *C. gloeosporioides* GH17 family genes for the recognition of host plant surface signals.

**Figure 3:**
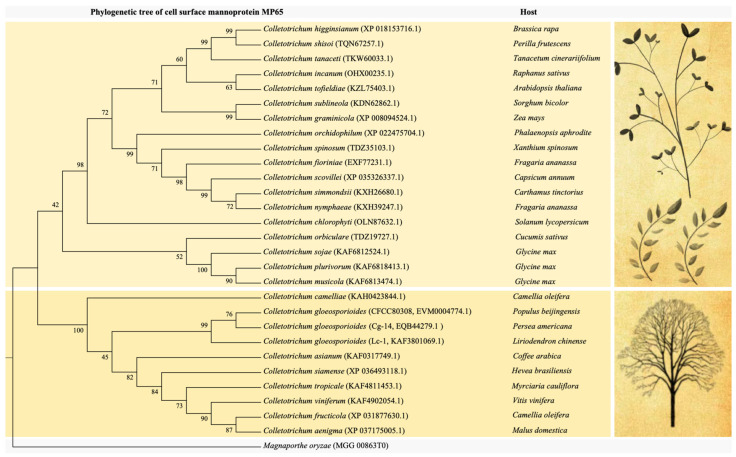
Phylogenetic tree of cell surface mannoprotein MP65-encoding genes in different *Colletotrichum* species and *Magnaporthe oryzae* as an outgroup.

We constructed a phylogenetic tree for MP65 (EVM0004774), which is a GH17 family protein associated with glycosylphosphatidylinositol (GPI)-anchor biosynthesis (ko00563) (Figure 3). The phylogenetic tree was constructed on the basis of the aligned MP65 sequences from *C. gloeosporioides* strains CFCC80308, Cg-14, and Lc-1, as well as other *Colletotrichum* species that can infect herbs and woody plants. The MP65 proteins from various *Colletotrichum* species were divided into two broad clades (Figure 3). The CFCC80308, Cg-14, and Lc-1 MP65 sequences were similar to the MP65 (KAH0423844) sequence in *Colletotrichum camelliae*, which can infect *C. oleifera*, suggestive of additional molecular differences. These findings reflect the expansion of the MP65-encoding gene family during the adaptation of *Colletotrichum* species to woody plants. Hence, gene gains and losses are important evolutionary processes for the adaptation of organisms to their environment.

### 3.4. Candidate Genes in Expanded Glycosyde Hydrolase Families

**Figure 4:**
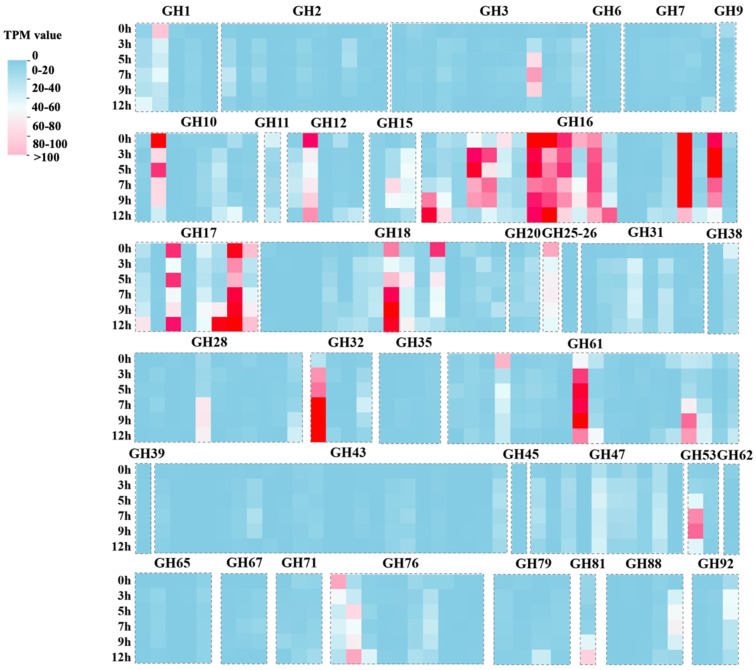
Differentially expressed glycosyde hydrolase (GH) family genes in *Colletotrichum gloeosporioides* strain CFCC80308 among the selected time points (0, 3, 5, 7, 9, and 12 h).

To clarify whether the expansion of the GH families in *C. gloeosporioides* influenced the adaptation of this phytopathogenic fungus to poplar trees, we inoculated *P. beijingensis* leaves with *C. gloeosporioides* strain CFCC80308. The transcriptome data indicated the expression levels of the genes in the GH1, GH3, GH10, GH12, GH15, GH16, GH17, GH18, GH25, GH32, GH53, GH61, GH76, and GH81 families increased substantially during the infection process (Figure 4). Notably, the expression levels of more than 62% of the GH16/GH17 family genes were significantly up-regulated at all time points (Figure 4, Table S3), and the MP65 gene (EVM0004774) was highly expressed at 9 and 12 h (Table S3), which was consistent with the qRT-PCR results of 3.3 (Figure 2). It was suggested these genes may be important for *C. gloeosporioides* to recognize surface signals of poplar trees.

## 4. Discussion

During evolution, gene duplication events may result in genes with novel functions [5]. Gene family expansions are reportedly crucial for adaptive evolution [50,51]. In the present study, a comparison of the genomes of *C. gloeosporioides* strains CFCC80308, Cg-14, and Lc-1 revealed the expansion of multiple gene families, with possible implications for the adaptation of *C. gloeosporioides* strains to woody plants. Several earlier studies examined *C. gloeosporioides* genomes [17,22,23]. In addition, extensive gene losses and gains, gene family expansions, and the evolution of novel genes have been reported for *Colletotrichum* strains [14,15,52]. However, the genes related to woody plant surface signal recognition and adaptation have not been thoroughly characterized. In this study, we analyzed the genomes of three *C. gloeosporioides* strains and detected the expansion of many GH gene families with potential roles during the recognition of plant surface signals. In addition to poplar trees, several tree species (e.g., *J. regia*, *M. indica*, *H. brasiliensis*, *C. oleifera*, and *C. lanceolata*) are susceptible to *C. gloeosporioides* infections, which result in rotted leaves, branches, or fruits. There are considerable differences in the chemical signals on the surface of plants, which may help to explain the diversity in the mechanisms by which *C. gloeosporioides* recognizes these signals.

The genetic diversity among *C. gloeosporioides* strains that infect different host plants may be related to the required adaptation to the plant surface chemical signals, which vary between plants. We detected changes in the number of genes in the families mediating the recognition of chemical signals and xenobiotic detoxification pathways. To successfully infect the host, *C. gloeosporioides* must first penetrate the plant epidermal tissue, suggesting the expanded gene families (e.g., GH families) may have influenced the adaptation to new hosts. Most of the genes related to plant cell wall degradation encode GHs. The pathogenicity of *C. gloeosporioides* may be related to the fact it produces many GHs when infecting susceptible plants [53].

To screen for *C. gloeosporioides* genes contributing to plant surface signal recognition, we compared the genomes of three *C. gloeosporioides* strains. The fungal cell wall is mainly composed of carbohydrate polymers and glycoproteins. The conserved carbohydrate components within the fungal cell wall include chitin, β-glucan, and mannan [54], which tend to be located in the inner, middle, and outer parts of the cell wall, respectively. Thus, mannan covers β-glucan and chitin, thereby impairing the ability of plants to perceive phytopathogenic fungi. Glycosyde hydrolases are key enzymes for degrading the aforementioned polysaccharides [55]. Many GHs that hydrolyze different substrates originated from the same ancestor and have similar structures and functions [56]. The results of the current study suggest a significant expansion of GH gene families occurred during the adaptation of *C. gloeosporioides* to woody plants. In addition, the expression of the genes in the GH1, GH3, GH10, GH12, GH15, GH16, GH17, GH18, GH25, GH32, GH53, GH61, GH76, and GH81 families increased substantially as *C. gloeosporioides* infected poplar leaves. The GH18-related enzymes can convert chitin to chitosan or N-acetyl glucosamine. In addition, glucan endo-1,3-β-glucosidases in the GH17 family contribute to starch metabolism, while β-1,3-glucan is converted to glucose. Therefore, the *C. gloeosporioides* GH17 family may play a key role in the adaptation with woody plants (Figure 4, Table S3).

The cell surface protein MP65 in the GH17 family is one of the main components of the fungal cell wall. Mannans are formed by the glycosylation of specific proteins. The initial glycosylation usually takes place in the endoplasmic reticulum, with the resulting protein subsequently processed in the Golgi apparatus [57]. There may be major differences in the mannose-based patterns among fungal species. Heterogeneous mannose glycosylation may occur in the strains and morphological types of a single species [58]. We speculate that MP65 in *C. gloeosporioides* is the result of the long-term evolution of specific GHs that are mainly involved in glycosylphosphatidylinositol (GPI)-anchor biosynthesis (ko00563, Figure S1) and glycerophospholipid metabolism (ko00564, Figure S2). Furthermore, the *Colletotrichum* gene family that includes the MP65-encoding gene probably expanded as *Colletotrichum* species co-evolved with their host plants, which indicates that gene gains and losses are important evolutionary processes that allow organisms to adapt to their environment (Figure 3). However, most of the hemicellulose-degrading enzymes (e.g., β-xylosidase) in fungi belong to the GH3 family [59,60]. Earlier research confirmed that β-xylosidase degrades xylooligosaccharide into xylose [61]. After the initial interaction between the pathogen and the host plant, the secretion of plant-cell-wall-degrading enzymes helps the pathogen penetrate the plant’s surface. The up-regulated expression of GH-encoding genes is important for the hemibiotrophic lifestyle of *C. gloeosporioides*.

A previous study showed that GH-encoding genes are overexpressed when the hemibiotrophic fungi causing anthracnose shift to a necrotrophic lifestyle [53]. The interaction between *C. gloeosporioides* and host plant leaves triggers the localized expression of genes encoding specific cell-wall-degrading enzymes, including cutinases, cellulases, and pectin lyases. The conidia of *C. gloeosporioides* recognize various host-induced signals (e.g., enzymes, hydrophobicity, surface hardness, leaf wax, and keratinocytes). The resulting induction of downstream signal transduction pathways leads to the formation of the appressorium that penetrates the epidermis of the host plant leaves, enabling the infection to progress.

In conclusion, the expression levels of GH family genes were highly up-regulated during the infection of poplar trees, which facilitated the recognition of the surface chemical signals of various woody plants. We propose that the evolution and expansion of these gene families enhanced the ability of *C. gloeosporioides* to perceive plant surface signals and overcome host defenses. These findings have contributed to elucidating the adaptation of *C. gloeosporioides* to woody plants.

## Figures and Tables

**Table 1:** Genome assembly details for three *Colletotrichum gloeosporioides* strains.

Strain Code	CFCC80308	Cg-14	Lc-1
Host	*Populus * *beijingensis*	*Persea * *americana*	*Liriodendron * *chinense*
Assembly size (bp)	60,701,134	53,209,944	61,904,035
Scaffolds/Contig	214	4537	128
N50	5,253,792	25,337	709,944
Genes	16,075	16,538	15,744
BUSCO coverage (%)	98.62	90.7	99.1
BUSCO single copy (%)	98.28	90.5	98.8
Reference	This study	[22]	[23]

**Table 2:** Analysis of *Colletotrichum gloeosporioides* protein families.

Strain Code	CFCC80308	Cg-14	Lc-1
Cytochrome P450	215	196	216
GH	368	343	365
CAZy	19	19	18
GPCR	7	7	7
Dehydrogenases	6	5	5
Transcription factors	569	481	560
SNARE proteins	10	10	10
CFEM proteins	34	31	34
SH3 proteins	4	4	4
HET proteins	190	141	190

## Data Availability

Data are contained within the article or Supplementary Materials.

## Supplementary Materials

The following supporting information can be downloaded at https://www.mdpi.com/article/10.3390/jof9121185/s1. Table S1: Classification and statistics of gene families; Table S2: Statistics of specific genes; Table S3: Differential expression genes of glycosyde hydrolase family (GHs) of *Colletotrichum gloeosporioides* CFCC80308 strain in different time intervals; Figure S1: Glycosylphosphatidylinositol (GPI) -anchor biosynthesis (ko00563); Figure S2: Glycerophospholipid metabolism (ko00564).
