# Supplementary material for: Gene Family Expansion during the Adaptation of Colletotrichum gloeosporioides to Woody Plants
Source: J Fungi (Basel). 2023 Dec 11;9(12):1185. doi: 10.3390/jof9121185 (PMC10744947; doi:10.3390/jof9121185)
Supplement: Supplementary file 1 [file jof-09-01185-s001.zip › jof-2751608-supplementary.pdf]

Table S1 Classification and statistics of gene families

Species Name	Total Gene Number	Cluster Gene Number	Total Family Number	Unique Gene Family Number
CFCC80308	16,075	15,261	14,251	11
Cg-14	16,538	13,955	12,845	4
Lc-1	15,672	15,038	14,073	5

Table S2 Statistics of specific genes

Species Name	Share Gene Number	Species Unique Gene Number
CFCC80308	15,226	849
Cg-14	13,947	2,591
Lc-1	15,027	645

Table S3 Differential expression genes of glycosyl hydrolase family (GHs) of *Colletotrichum gloeosporioides* CFCC80308 strain in different time-points

Family	Gene ID	0h	3h	5h	7h	9h	12h
GH1	EVM0004161	5.575843	14.011963	14.6220773	24.7674727	21.2526227	43.596584
	EVM0012359	90.8156475	37.826763	32.392165	44.504518	26.7575397	24.497097
	EVM0001457	2.716639	1.0330005	0.85183567	0.58878467	0.649663	2.061501
	EVM0010403	0.15368	4.001115	4.89906567	5.08891133	4.342746	4.88073
	EVM0011391	0.118866	0.1729705	0.47805133	0.947028	1.07801033	1.105943
GH2	EVM0003176	1.7179155	10.9813795	12.1166373	26.6787567	19.623855	4.540607
	EVM0006253	0.0115105	0.1540115	0.30562133	0.61156167	0.29576933	0.104657
	EVM0008383	0.1899925	7.774685	5.80241267	12.6949647	5.87929633	3.258103
	EVM0012095	0.030274	0	0.07989567	0.050575	0.07136167	0.274304

	EVM0000421	0.11004	0.5208875	0.34715233	0.821769	0.61456167	0.969967
	EVM0002091	0.555576	6.167673	4.222014	8.21396467	4.212885	1.677679
	EVM0007833	2.0702565	1.017687	1.34812967	1.937151	1.62139633	0.639287
	EVM0006176	0.0356075	0.012677	0.132599	0.07931533	0.19841433	0.287484
	EVM0006024	0.469871	15.809944	18.5400803	6.44587067	3.99710533	0
	EVM0013277	0.226119	1.658443	1.23023867	1.90347067	0.83430267	0.606327
	EVM0009128	1.913509	0.379218	0.52077067	0.34140967	0.636684	0.22464
GH3	EVM0004149	0.9654825	0.4504775	1.04281033	1.09940667	0.89598267	1.224844
	EVM0009235	5.8767265	2.9926125	2.004359	3.789533	2.24230667	2.462648
	EVM0008949	2.144027	1.6540425	1.631668	1.557396	1.21342467	0.90387
	EVM0007160	0.2270795	7.5784345	6.257062	11.8021407	4.925589	5.112597
	EVM0013736	1.4362135	5.1759835	2.593537	3.692907	2.677861	0.593943
	EVM0003342	0.3055315	0.319085	0.70444433	0.67447967	0.79408067	0.362579
	EVM0003379	0.140213	2.0052275	1.28182533	2.27523867	1.49113133	1.161446
	EVM0002132	0.0807075	1.684209	1.19015733	3.61992967	2.629284	0.651292
	EVM0013369	0.172183	1.136255	1.99066567	4.71670567	2.02244633	1.30091
	EVM0013323	1.4902465	21.5015775	73.2580007	120.261225	80.4821347	7.038403
	EVM0006514	1.3870805	2.9785255	0.99494467	2.08223	2.92792567	2.886358
	EVM0000962	0.0949795	6.418158	4.16763467	5.94645867	2.86447433	2.106311
	EVM0012829	3.152678	17.8577565	13.7615343	16.811614	14.9002857	7.873043
GH6	EVM0002940	0.120472	0.057562	0.15079567	0.11421733	0.09454867	0.121564
	EVM0010314	1.359525	2.1399695	2.782551	2.45320167	2.262287	1.235268
GH7	EVM0005737	0.1852735	0.563144	0.58965967	0.94331367	0.62832733	0.197818
	EVM0012022	0.7742225	2.5907625	2.623519	2.97222567	2.21298833	1.37894
	EVM0010243	0.402505	2.8085165	2.36337733	2.34491167	2.10700733	0.645552
	EVM0008745	1.4392065	4.031551	3.43492733	3.87947267	2.16310067	3.80516
	EVM0004977	2.2172855	3.4848115	7.04356	2.682563	1.63482767	0.14321
	EVM0003261	5.974459	0.1247605	0.05177133	0.146008	0.585579	14.851276
GH9	EVM0009880	14.10007	1.8057025	4.009368	3.19466767	4.76849967	2.984877
GH10	EVM0005978	5.5424675	0.228794	3.42894567	1.93543667	0.90392033	0.249006
	EVM0000486	583.792084	76.672638	187.870244	76.6636467	75.5301303	33.962406
	EVM0009108	0.738452	0	0.43834433	0.41378567	0.355133	1.024382

	EVM0000483	2.860495	0.744519	0.62186867	1.490053	1.751228	0.957239
	EVM0006517	2.6473345	5.8010345	10.2480723	10.4591457	9.17996867	3.401612
	EVM0001473	2.1440095	20.202843	28.797853	24.0059457	20.2761507	31.315237
	EVM0001249	16.82504	1.6096305	1.93197667	1.02489467	6.20489267	45.282284
	EVM0012819	2.793963	2.5577355	4.52302067	3.17626767	3.24542967	5.584347
GH11	EVM0006462	37.4588555	10.87578	8.43714433	13.4083383	10.7589853	27.615372
	EVM0015211	2.169069	15.023395	26.395183	30.4825047	24.1639633	2.689664
	EVM0009853	204.822991	61.000561	52.278547	63.2392577	84.1420037	128.475571
GH12	EVM0005727	1.5029665	0.5938035	0.793579	1.49296967	0.90333733	0.725076
	EVM0008944	18.8335195	2.4197835	3.409614	2.84006233	4.952283	18.730154
	EVM0004416	1.309679	1.819672	1.46929567	2.17633833	2.23040033	28.470934
	EVM0003981	3.493362	3.6830375	4.13701167	3.77086233	4.37136333	4.202559
GH15	EVM0005982	0.72342	16.9426985	16.834846	75.7022513	52.1722267	6.41205
	EVM0003549	6.23553	45.1551515	51.5938427	41.9912923	46.662103	18.417475
	EVM0012540	2.3624085	5.428973	3.05412167	20.610491	141.969367	249.077148
	EVM0003099	20.062954	12.5294355	13.165674	30.7518697	49.101194	72.893127
	EVM0011758	14.764948	7.850862	5.198039	5.35859633	5.40331567	16.543509
	EVM0000635	54.074482	207.498005	256.649083	111.081335	122.91065	13.261082
	EVM0008145	29.5703615	178.425545	67.8334707	153.224744	134.941733	41.725857
	EVM0012239	69.6815185	34.3559885	39.079376	15.171675	22.6824483	26.59738
	EVM0013723	22.9508125	12.0364555	8.05208567	13.4772347	12.0511737	22.322897
	EVM0005991	1558.85321	233.816285	251.339498	145.222176	198.655894	209.730057
GH16	EVM0006710	863.396973	112.369976	140.652476	155.368612	178.814265	466.735107
	EVM0009199	192.105965	171.888527	188.995606	101.219663	130.645943	79.713264
	EVM0016050	106.7327	13.915598	17.1217777	51.9252447	64.118552	36.62439
	EVM0006293	130.190232	167.257713	177.421346	178.906942	158.976102	103.390068
	EVM0014972	19.275485	15.261852	28.5655627	24.6682657	40.1929143	165.143845
	EVM0001057	0.0361655	0.317747	0.17373067	0.653294	0.285779	0.166725
	EVM0004431	1.1367905	1.090165	0.74739033	1.328857	0.99433233	0.938059
	EVM0010803	1.8572845	1.262971	0.41896267	1.40520133	5.756054	24.011557
	EVM0002247	8.036932	9.2537885	9.83647133	14.368782	15.3805153	19.466375
	EVM0010284	563.997803	501.160584	438.958816	368.828461	349.021479	98.719223

	EVM0015596	1.442928	6.1827995	9.65128167	3.937384	8.707249	35.253845
	EVM0001285	221.972794	337.334023	301.102809	162.613899	134.800611	53.007576
	EVM0015862	9.7760215	2.601666	0.51364367	1.062766	1.67849	5.509372
GH17	EVM0015671	21.893778	11.683155	22.6613297	24.2127207	31.78502	67.824356
	EVM0001170	0.663265	1.269865	1.27327233	1.86470933	1.13892967	1.269363
	EVM0004026	190.117112	49.709585	188.025645	52.902874	68.638954	200.330566
	EVM0012289	2.4015365	2.127532	2.57133567	1.61975167	1.653873	3.360688
	EVM0005681	39.0738975	24.4925135	23.381495	35.54482	53.4055367	47.753353
	EVM0004774	16.2282545	14.5730775	8.45233967	10.325291	62.5091987	271.992126
	EVM0010167	269.372078	132.33297	104.371399	240.141113	264.408137	296.048859
	EVM0004080	95.869055	24.766924	17.0049153	42.419085	61.4844983	95.072662
GH18	EVM0002948	2.793026	0.2680585	0.07987233	0.01735533	0.02730033	0.790769
	EVM0003071	0.0490585	0.6042635	0.21825533	0.27524267	0.16726967	0.368595
	EVM0003067	0.137785	0.090539	0.155518	0.18885	0.18452933	0.25202
	EVM0010267	0.1713275	0.229368	0.36368633	0.07889633	0.05063333	0.039984
	EVM0001037	2.3827715	7.115821	11.34431	15.3493757	9.97768433	9.451127
	EVM0000981	0.6437415	0.659101	0.71502533	1.481891	8.0725	16.435019
	EVM0004848	3.081653	21.160327	15.935952	16.2991853	15.007039	21.625706
	EVM0008318	9.6812745	23.5734055	17.7160043	26.027253	27.4665363	10.471504
	EVM0009169	141.777414	46.734093	97.660192	220.320994	440.730855	257.925934
	EVM0010724	10.795301	33.4687025	62.552957	36.679285	37.093493	58.040314
	EVM0012702	4.6846275	4.852484	3.11811733	7.785705	9.53691033	26.322693
	EVM0009586	188.86287	27.9192065	63.8753613	46.5848643	51.9091847	20.0345
	EVM0007051	0.850959	0.2421645	0.13887967	0.46136367	0.48746033	5.91629
	EVM0010233	11.828802	3.2240755	3.87975367	5.157439	4.49827267	4.074782
	EVM0012316	5.4749525	22.207528	8.79351733	13.7864907	7.113948	2.997464
	EVM0014669	0	5.3161435	8.791847	4.095918	22.5868837	4.608161
GH20	EVM0007680	0.892951	4.665193	8.894116	7.32022467	11.1125367	2.56951
	EVM0011676	6.060044	18.8372955	14.7936517	15.8137343	10.7959853	5.397075
GH25	EVM0004167	111.635086	44.2942065	59.6817537	61.8492583	55.0380047	44.86631
GH26	EVM0015995	0.697464	0.223636	0.15091067	0.10221633	0.24851133	1.08506
GH28	EVM0002541	0.0168785	0.4426915	2.12038933	0.71045333	0.60353033	0

	EVM0012026	0.2801915	3.6523325	2.08610333	6.17664933	2.47115433	0.702962
	EVM0007701	1.3885575	0.284853	0.13332267	0.231108	0.06496	1.019111
	EVM0001669	0.1007795	0.9349575	0.97912667	1.00454367	0.68750733	0.439062
	EVM0001425	12.533371	10.6827855	12.22998	64.876326	66.3982467	60.26495
	EVM0016027	0.457841	4.7456165	2.74649267	1.98133867	1.17518967	0.235594
	EVM0012933	1.3678395	1.60159	1.21212633	1.018709	1.18987033	1.880633
	EVM0015584	4.126273	1.142255	1.30054433	2.19063867	1.61765	3.581266
	EVM0004478	0.886273	1.5149115	1.283088	1.22697767	0.706564	0.979487
	EVM0005577	0.7821475	1.4772515	1.92026567	3.36878467	2.64073333	0.418388
	EVM0008421	12.5781595	9.537998	4.647559	8.23807567	25.8589303	34.406635
GH31	EVM0010580	0.0400875	0.625505	0.47882533	0.50929133	0.345326	0.060026
	EVM0000329	0.678298	10.458913	8.06043567	9.18457333	4.68875967	3.89494
	EVM0013931	0.852896	13.5065925	7.43176667	10.629525	10.870236	14.455359
	EVM0004264	2.9126385	37.9484025	31.7366637	37.1549747	34.47084	24.343224
	EVM0015745	1.269854	2.682721	1.73304533	1.36174233	2.33706967	5.880241
	EVM0006474	1.3303935	22.44716	12.873013	26.144661	24.046705	24.469347
	EVM0010510	0.055955	2.29713	1.02078433	1.05193133	1.35247233	0.364311
	EVM0002802	0.511566	1.2909625	1.68530033	4.17398433	1.88443667	0.882815
GH32	EVM0010355	23.760446	126.906611	147.538149	299.210709	541.768382	639.033691
	EVM0014483	1.8665885	4.144883	0.68057533	0.40816867	0.93199733	6.71406
	EVM0007614	0.2745345	0.270601	1.31544933	0.448612	0.76378067	0.524607
	EVM0011322	2.40376	11.064781	18.8569047	34.6874047	17.357674	6.738147
GH35	EVM0012242	0.396719	0.489289	0.41547333	0.317993	0.13758967	0.550332
	EVM0000434	0.054976	0.9056835	0.88343233	2.70984533	2.37141933	0.652609
	EVM0008711	0.5676705	1.1852635	0.801759	1.510612	0.78329933	0.565181
	EVM0001099	2.266324	2.9817485	3.33357067	2.09280633	1.33261	1.732914
GH38	EVM0009804	0.0408885	0.0861665	0.06439	0.09430033	0.07695033	0.042883
	EVM0004698	38.9512425	17.604556	12.316683	11.4740883	10.63269	12.966511
GH39	EVM0015791	0.31269	0.6435105	1.78612967	1.33708867	1.13020533	0.177111
GH43	EVM0003464	0.4007975	3.3764185	4.05479333	6.07021967	3.59527767	2.871799
	EVM0001144	1.2700475	0.1344605	0.070375	0.18006633	0.23780767	0.456385
	EVM0006438	0.0539025	0.1068005	0.450274	0.40063033	0.26127433	0.127765

	EVM0006945	0.0893485	0.2394855	0.27956733	0.160301	0.06306467	0.054377
	EVM0006521	0.167094	0.8615865	0.38269967	0.51882267	0.652484	0.256446
	EVM0000948	0.7065785	2.4972075	2.81325633	4.19897233	3.68762267	1.503418
	EVM0001154	0.0642155	9.243209	9.185183	19.717071	14.999233	0.105439
	EVM0011361	1.412442	0	0	0.03179633	0.15686133	2.741227
	EVM0015608	0.057858	0.336229	0.99641067	1.62012433	0.681365	0.170323
	EVM0004345	3.4251815	2.528313	5.23617033	3.573909	2.49397933	2.857158
	EVM0002892	3.7553115	3.9977285	3.313034	4.771385	6.351048	2.034461
	EVM0009597	0.047962	0	0.04752367	0.16572767	0.09639167	0.094892
	EVM0002546	0.124969	0.191293	0.27806167	0.33205733	0.24856333	0.142575
	EVM0007240	0.828748	0.6568555	1.99270633	3.435839	2.380416	0.384478
	EVM0007731	0.862593	0.6229755	0.30667567	0.48792933	0.609914	0.292219
	EVM0006952	0.7156805	3.708741	6.46883967	5.997616	6.563617	5.07761
	EVM0001225	0.5195995	7.105398	7.35391133	11.669322	6.17272233	11.198683
	EVM0002856	0.1181045	0.2477785	0.38846133	0.30968467	0.62831533	0.298429
	EVM0005044	1.594639	2.471537	1.32612433	3.06317067	2.76988467	2.288375
	EVM0010891	0.226193	0.0090335	0.118294	0.22732533	0.12637067	0.457046
	EVM0003879	0.154003	0.0905435	0.30076133	0.38758733	0.542843	0.225183
	EVM0010214	0.4478875	0.369751	0.29692533	1.21860233	0.788085	1.063476
	EVM0005108	5.628318	15.357228	19.4405047	12.929865	15.871662	12.006785
GH45	EVM0001050	0.3126375	0.4243235	1.410067	1.16351967	0.908767	1.199167
GH47	EVM0012513	2.284885	15.925919	15.5387817	20.5163413	15.114749	8.348307
	EVM0008209	0.1533455	1.2640425	1.24620867	2.21120267	1.36287633	0.378245
	EVM0001485	8.1928565	15.5759045	14.7076867	12.3588377	12.113689	14.801183
	EVM0011574	0.3942675	0.490082	0.45719533	0.303191	0.25236933	0.477304
	EVM0014924	18.9779425	32.810433	38.1353863	32.7489863	33.8478267	36.442699
	EVM0006394	3.9143655	16.5589725	19.83771	15.4839957	13.633725	7.374712
	EVM0008767	11.455677	16.7004365	17.8528127	15.3197273	14.6806047	7.828476
	EVM0014538	0.731372	5.09601	3.62749933	4.25663467	4.89013767	4.971745
	EVM0000430	18.6626125	18.350547	25.1609257	29.0375897	28.752808	23.32596
	EVM0014030	0.275719	0.103789	0.13594633	0.154162	0.50272833	0.375847
GH53	EVM0015635	3.183965	9.1216555	43.4137183	134.155645	157.138842	42.081955

	EVM0001294	1.3419455	5.7421445	4.74623233	4.21214767	2.71248033	1.286906
	EVM0014169	2.375887	3.3083755	10.1243627	3.60090733	4.55635167	0.208806
	EVM0014803	6.16441	7.5617095	5.71204333	5.164329	5.55754033	9.579249
	EVM0000498	0.8182365	4.0866775	3.559352	1.62641967	3.04386067	4.341513
	EVM0002763	103.438836	21.0937175	45.4696233	36.919373	29.3699927	28.728249
	EVM0007125	1.0225825	2.7781455	5.777932	4.35124267	5.21586167	1.526566
	EVM0014344	2.4299245	8.4128545	10.1676607	5.39142233	6.81689267	2.446816
	EVM0013798	3.4573885	4.854521	6.14835367	3.09935767	2.95194733	0.456597
	EVM0013315	1.4969195	0.295533	0.355848	1.33398533	1.59149633	0.47057
	EVM0011511	54.4149015	182.524689	248.4746	230.481712	321.354622	138.567368
GH61	EVM0011977	24.74261	5.1528615	4.83882833	9.585877	13.8467867	53.308594
	EVM0009384	3.242511	2.243838	2.98351767	1.00761433	2.03984667	2.368871
	EVM0005936	0.033309	0.403723	0.98100267	0.80716733	1.52825167	0.285083
	EVM0011907	0.8142065	3.1103565	1.73465	0.27115233	0.39819133	0
	EVM0000452	2.8052415	0.868342	1.09499067	1.83716933	2.46175933	8.775876
	EVM0005869	18.315985	0.34297	0.36045733	0.20191733	0.50187967	22.272444
	EVM0011109	28.045839	10.1314915	15.0506513	60.9535347	153.878912	122.103348
	EVM0001634	24.735445	5.878322	5.591528	17.3928733	33.1081867	37.526077
	EVM0009521	4.7963825	0.1556365	0.15780433	0.571929	2.14308467	5.448975
	EVM0007129	14.114715	6.436733	5.21477067	9.95420367	13.3771673	28.698503
GH62	EVM0000346	2.624409	0.0967285	0.35929367	0.47536333	0.34743433	0.579507
	EVM0008572	0.248576	1.982955	0.88923367	1.11048733	0.44873033	0.457284
	EVM0003568	0.334348	6.931783	5.83241567	6.40894933	5.36790867	1.431793
GH65	EVM0006667	0.0535205	0.1273985	0.11588067	0.404116	0.33821433	0.438866
	EVM0009640	0.05376	0.5261495	0.16808767	0.48292767	0.45730633	0.154048
	EVM0013270	3.582705	8.2072635	4.73329333	5.23578467	6.00867	5.10638
	EVM0012117	0.442662	1.13919	0.73512533	1.24616567	1.31823667	0.65878
GH67	EVM0001966	0.045518	1.736851	1.896452	5.42324367	2.95282967	0.077498
	EVM0007149	0.2286455	3.5947325	4.04895833	9.16705667	4.083379	1.179614
	EVM0014343	0.056321	1.602128	2.920992	3.426869	9.66310433	1.243625
GH71	EVM0005159	7.861872	2.5760025	3.10733333	2.142394	6.01584	8.071768
	EVM0007849	6.504255	2.7931325	3.548622	6.08702467	7.53525167	14.297135

GH76	EVM0000572	113.56419	52.9828815	32.0068493	39.0157633	31.8027433	21.506603
	EVM0001565	15.8726065	29.9345875	77.5162683	50.8740233	61.2646613	112.866173
	EVM0012197	2.661719	2.7557355	8.03007133	2.847825	7.59863367	40.621956
	EVM0009284	1.345416	1.9449395	0.82098633	1.22383	0.784619	1.842289
	EVM0015731	0.6827605	2.4777065	1.82187767	1.43657567	1.18546767	1.352388
	EVM0006413	10.4754165	4.5796595	6.81655667	9.406344	10.0905943	18.730944
	EVM0010626	5.757337	19.902379	24.4762663	30.1620917	25.2169567	14.154141
	EVM0009713	5.680979	0.9497535	0.70850167	0.660055	0.646035	0.955415
	EVM0012093	0.601274	0.6011425	0.743221	0.48648367	0.88641867	0.622133
	EVM0005755	0.961253	0.2953565	0.30747767	1.071287	1.04665267	1.506657
GH79	EVM0007644	1.6855385	5.083116	5.04443133	4.68146033	4.11441133	2.190292
	EVM0007287	2.6831755	0.834942	0.84686	1.37738367	1.67666433	2.826605
	EVM0012154	0.753332	1.1731765	2.21731467	3.82637367	5.45541967	31.269463
	EVM0011877	1.339445	3.869463	4.94445033	4.814094	4.46877233	2.123093
GH81	EVM0000425	8.2025305	7.6344555	3.18659967	5.33214433	43.1716873	81.985298
GH88	EVM0009009	0.3917155	0.4986395	0.477335	0.28059233	0.317514	0.175774
	EVM0013244	0.9936165	0.516833	0.17030033	0.28492067	0.36132333	0.388692
	EVM0013773	0.212512	0.074819	0.033041	0.49121067	0.579345	1.076711
	EVM0013092	4.094452	2.8352575	4.89628733	3.59346333	4.68346067	11.817643
	EVM0012554	1.294482	36.6580725	54.0429097	59.0606487	37.506266	10.0333
GH92	EVM0001240	0.5787005	1.5216765	0.817008	1.07749867	0.550375	0.882574
	EVM0002670	1.2661435	3.5610875	2.70887533	2.83912633	2.55903867	2.46198
	EVM0010947	14.793209	48.513714	47.1735787	24.355355	24.0019943	16.277323

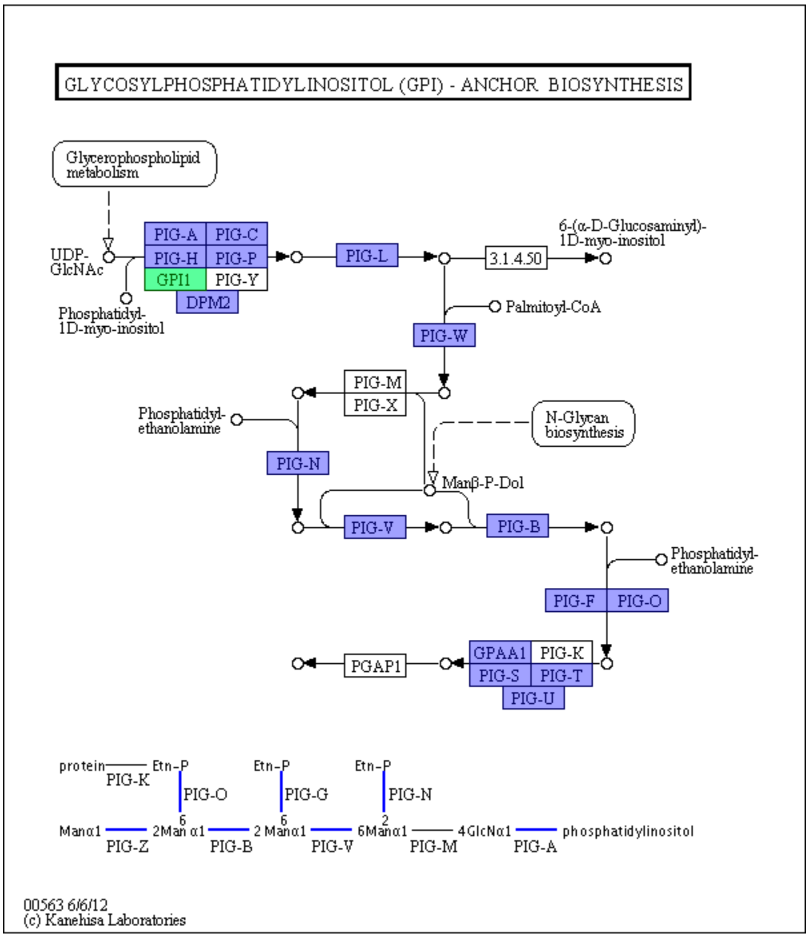

Figure S1 Glycosylphosphatidylinositol (GPI) -anchor biosynthesis (ko00563)

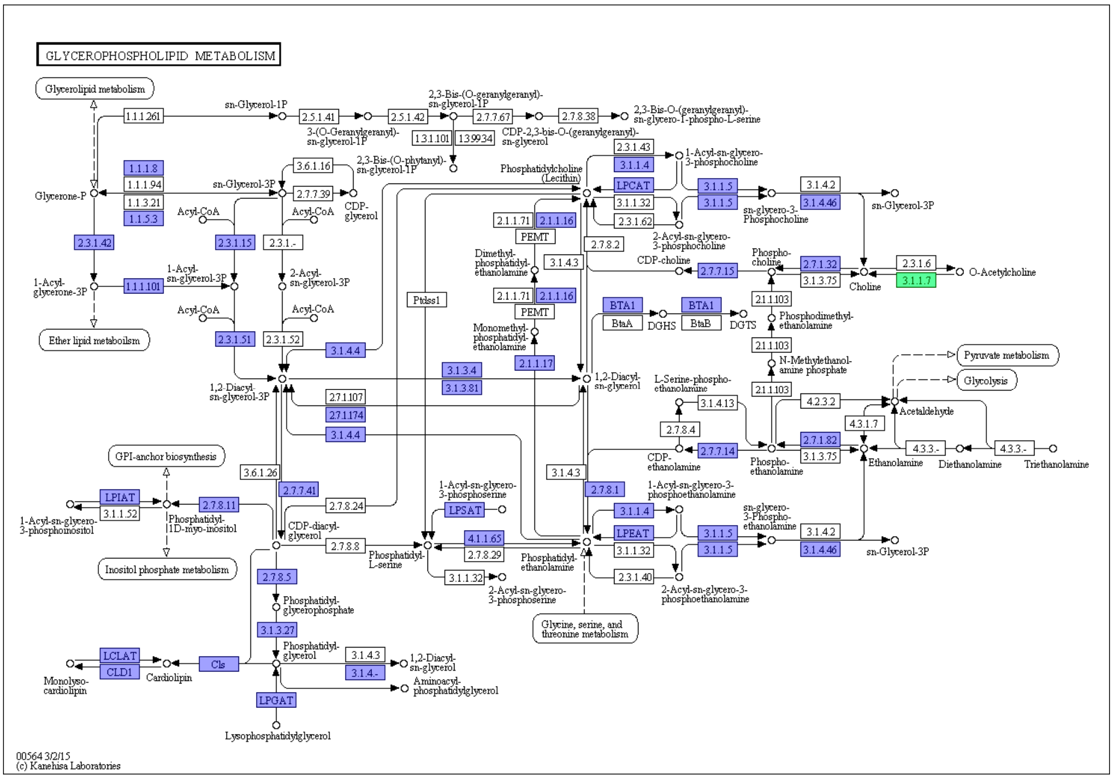

Figure S2 Glycerophospholipid metabolism (ko00564)
